# EZH2 overexpression in different immunophenotypes of breast carcinoma and association with clinicopathologic features

**DOI:** 10.1186/s13000-016-0491-5

**Published:** 2016-04-26

**Authors:** Shuangping Guo, Xia Li, Joseph Rohr, Yingmei Wang, Shirong Ma, Peng Chen, Zhe Wang

**Affiliations:** Department of Pathology, Xi Jing Hospital, the Fourth Military Medical University, Xi’an, Shaan Xi Province 710032 China; Department of Pathology and Microbiology, University of Nebraska Medical Center, Omaha, 68105 NE USA

**Keywords:** EZH2 overexpression, Immunophenotype, Triple-negative breast carcinoma, Proliferative index, High nuclear grade

## Abstract

**Background:**

Enhancer of zest homolog 2 (*EZH2*), a histone 3 methyltransferase, is associated with aggressive behavior of many tumors and is a promising target of molecular therapy.

**Methods:**

To better elucidate the relevance of EZH2 in breast cancer subtypes, we evaluated EZH2 expression in 226 invasive breast carcinomas with four distinct immunophenotypes and in association with clinicopathological features.

**Results:**

Of these cases, 138 (61.1 %) were defined as EZH2-overexpressing with a multiplicative score > 3. EZH2 expression was inversely related to the status of ER and PR (Chi-square, *p* < 0.001), and it was significantly associated with HER2 positivity, high proliferative index, and high histologic grade (Chi-square, *p* < 0.05). ER-positive breast carcinoma with low proliferative index (Ki67 < 14 %) showed the lowest expression and triple-negative breast carcinoma showed the highest overexpression of EZH2, 18.5 % (10/54) versus 90.9 % (50/55) (Chi-square, *p* < 0.001). Intriguingly, 88 % (44/50) cases of grade 3 triple-negative breast carcinoma showed uniformly strong EZH2 expression with a multiplicative score of 9. The percentage of EZH2 overexpression in ER-positive breast carcinoma with a high proliferative index or HER2-positive cases were 61.2 and 74 %, respectively. Furthermore, EZH2 expression was significantly elevated in high-grade DCIS compared to benign lesions (90 % versus 0, *p* < 0.001). However, there is no association between EZH2 expression and the status of histone 3 lysine 27 trimethylation or other clinicopathologic features.

**Conclusion:**

In summary, triple-negative breast carcinoma showed the highest overexpression of EZH2. EZH2 overexpression is associated aggressive pathologic features including high nuclear grade, high proliferative index, and positivity of HER2 of breast carcinoma.

## Background

Breast carcinoma comprises an extraordinarily diverse group of diseases in terms of histology and clinical behavior and can be classified according to immunophenotypic features. ER-positive breast carcinoma is defined as ≥1 % of tumor cells being positive for ER, and usually these are also positive for PR [1]. HER2-positive carcinoma shows either protein overexpression detected by IHC or HER2 gene amplification by FISH [2]. Triple-negative breast carcinoma is defined as combined negativity for ER, PR and HER2, which constitutes about 20 % of breast cancer. Triple-negative carcinoma has attracted much interest as an aggressive group that does not benefit from the currently available anti-endocrine and anti-HER2 treatment [3–5]. It is necessary to identify a biomarker for treating this unique breast carcinoma [6, 7]. High expression of Enhancer zest homolog 2 (EZH2), a histone 3 methyltransferase, is related to unfavorable outcome to tamoxifen in metastatic breast cancer and is an independent predictor of survival in women with breast cancer [8, 9]. There are studies suggesting that EZH2 probably plays an important role in carcinogenesis of breast carcinoma [10–18], and high expression of EZH2 was associated with triple-negative breast carcinoma [15, 16, 18–21]. However, the association of EZH2 expression with clinicopathologic features such as PR, HER2 expression, nuclear grade, and proliferative index are inconsistent [8, 9, 22]. Reijm et al. reported that there was no significant association between EZH2 protein expression and menopausal status, tumor histology, PR status [8]. they found a significant positive association with the amount of lymph nodes involved, histologic grade and HER2 status instead [8]. Whereas, Bachmann et al. only reported high EZH2 expression was associated with high histologic grade, locally advanced cancers, and presence of distant metastatic disease of breast carcinoma [9]. However, Kleer et al. demonstrated that higher EZH2 levels were significantly associated with negative ER status, negative PR status, but not HER2 overexpression [22]. Furthermore, there is no reports concerning EZH2 expression in different immunohistochemical subgroups of breast carcinoma. So, in this study, EZH2 expression in 226 invasive breast carcinomas was assessed by IHC, and the association with clinicopathologic features was analyzed in Chinese cohort. We found that triple-negative breast carcinoma showed the highest percentage of EZH2 overexpression compared to ER-positive or HER2-positive subtypes, and 88 % (44/50) cases of grade 3 triple-negative breast carcinoma showed uniformly strong EZH2 expression with a multiplicative score of 9. Our study suggests that EZH2 overexpression is an immunophenotypic feature of high-grade and highly proliferative triple-negative breast carcinoma and is independently associated with a high proliferative index and HER2 positivity. Therefore, therapy targeting EZH2 should potentially be considered for triple-negative and high-grade HER2-positive breast carcinomas.

## Methods

### Patient materials

Two hundred twenty-six invasive breast carcinomas diagnosed between January 1998 and December 2014 were chosen from the archive at department of pathology in Xi Jing Hospital. The cases were reviewed by breast pathologists (S.P.G. and X.L.) and classified based on the World Health Organization criteria for breast carcinoma classification. The clinicopathologic information including patient age, menstrual status, tumor size, tumor stage, and lymph node stages were collected. The TNM staging was determined by the 7^th^ AJCC TNM staging system. The histologic grade of invasive breast carcinoma was graded according to the WHO criteria of breast carcinoma classification. 20 benign breast lesions comprising of adenosis and usual ductal hyperplasia (UDH), 20 DCIS adjacent to high grade triple-negative carcinoma and 20 DCIS adjacent to low to moderate grade non-triple-negative carcinoma were also assessed for EZH2. This study was approved by the institution review board of Xi Jing Hospital.

### Immunohistochemical staining and interpretation

The tissue was fixed in 10 % buffered formalin and processed as usual for paraffin embedding. Representative tissue blocks of invasive breast carcinoma were selected by examination of the corresponding hematoxylin & eosin (HE) stained slides and 5 μm thick sections were stained with rabbit anti-human EZH2 antibody. Immunohistochemical staining was performed using the Bond-Max automated immunostainer (Vision Biosystems, Lecia, Autralia), with a polymer-based detection system. EZH2 staining results was interpreted independently by two breast pathologists (S.P.G. and X.L.). Expression was assessed using a multiplicative score of intensity and percentage of cells stained for EZH2, where intensity (0 = no staining, 1 = weak, 2 = moderate and 3 = strong) and percentage (1 = <10 %, 2 = 11–50 %, and 3 = >50 %) were graded according to the method described previously [8, 9]. A multiplicative value of >3 was considered EZH2 overexpression and the maximum score of 9 was considered strong overexpression. Immunostaining data for estrogen receptor (ER), progesterone receptor (PR), human epidermal growth factor receptor 2 (HER2), Ki67, CK5/6, and CK14 were collected from the archive. ER, PR, HER2 were scored according to the ASCO/CAP guideline [1, 2]. The antibodies are shown in Table 1.

**Table 1:** The primary antibodies

Antibodies	Clone	Manufacturer	Dilution
ER	6F11	Ventana, Tucson, AZ, USA	Ready to use
PR	AB-52	Santa Cruz Biotechnology, Dallas, Texas, USA	1:100
HER2	Polyclonal	Sigma-Aldrich Shanghai Trading Co Ltd Shanghai, China	Ready to use
Ki67	MIB-1	DAKO, Denmark	Ready to use
CK5/6	D5/16B4	DAKO, Denmark	1:50
p63	4A4	Fuzhou Maixin, Biology, Fouzhou, China	Ready to use
CK14	LL002	Fuzhou Maixin, Biology, Fouzhou, China	Ready to use
EGFR	Polyclonal	Santa Cruz Biotechnology, Dallas, Texas, USA	1:100
EZH2	D2C9	Cell signaling Technology, Inc., Danvers, MA, USA	1:100
H3K27me3	C36B11	Cell Signaling Technology, Inc., Danvers, MA, USA	1:500

### Fluorescence in-situ hybridization

Thirty cases expressing a HER2 protein score of 2+ by IHC were assessed for the presence of HER2 amplification on 4 μm FFPE sections using a standard probe (Abbott Molecular, Des Plaines, IL). Cases were regarded as HER2 amplified if the HER2/CEP17 ratio was ≥2.0 or the average HER2 copy number ≥ 6.0 signals/cell [2].

### Statistical analysis methods

The associations between EZH2 expression and clinicopathologic features of the patients with breast carcinoma were analyzed using Pearson’s chi-square test. Statistical significance was defined as *p* < 0.05. Data were analyzed using the Statistical Package for the Social Science Version 17.0 (SPSS 17.0).

## Results

### The clinicopathologic features of breast carcinoma

All 226 patients were female with a median age of 47 (range 30–87) years who presented with breast masses. Mastectomy, or breast conserving surgery with negative margins and axillary lymph nodes excision were performed in all patients. Out of 226 cases, 26 were well differentiated invasive carcinoma of nuclear grade 1, 86 were nuclear grade 2, and the remaining 114 were poorly differentiated carcinoma of nuclear grade 3. All cases were invasive carcinoma, not otherwise specific, except ten tubular carcinomas, five mucinous carcinomas which were included in the group of nuclear grade 1. Immunostaining data for ER, PR, HER2 were collected from the archive. 121 cases were ER positive, and 105 cases were negative. 110 cases were PR positive, and 116 cases were PR negative. Out of thirty cases expressing a HER2 protein score of 2+ by IHC, only nine cases showed the presence of HER2 gene amplification. 50 cases were positive for HER2 protein or showed HER2 gene amplification, 176 cases were negative for HER2 according to the results of IHC and/or FISH in total. Furthermore, the tumor were classified into three groups according to tumor proliferation index Ki67 (<14 %, 14 %–30 %, >30 %), and the case number was 74, 73 and 79 in each group, respectively. The clinicopathologic information of the cases was summarized in Table 2.

**Table 2:** EZH2 expression in breast carcinoma and relationship with clinicopathologic characteristics

Clinicopathologic features		EZH2 score		
	Total N (%)	<3 score, negative	>3 score, positive	*P* value
Age at diagnosis (year)				
<50	133	36.8 % (49/133)	63.2 % (84/133)	*p* > 0.05
≥50	93	41.9 % (39/93)	58.1 % (54/93)	
Menopausal status				
Premenopausal	125	36 % (45/125)	64 % (80/125)	*p* > 0.05
Postmenopausal	101	42.6 % (43/101)	57.4 % (58/101)	
Estrogen receptor status				*p* < 0.001
Positive	121	57.9 % (70/121)	42.1 % (51/121)	
Negative	105	17.1 % (18/105)	82.9 % (87/105)	
Progesterone receptor status				*p* < 0.001
Positive	110	56.4 % (62/110)	43.6 % (48/110)	
Negative	116	22.4 % (26/116)	77.6 % (90/116)	
Her2				*p* < 0.05
Positive	50	24 % (12/50)	76 % (38/50)	
Negative	176	43.2 % (76/176)	56.8 % (100/176)	
Ki67				*p* < 0.001
<14 %	74	74.3 % (55/74)	25.7 % (19/74)	
14–30 %	73	31.5 % (23/73)	68.5 % (50/73)	
>30 %	79	12.7 % (10/79)	87.3 % (69/79)	
T stage				
T1	105	33.3 % (35/105)	66.7 % (70/105)	*p* > 0.05
T2	108	42.6 % (46/108)	57.4 % (62/108)	
T3	13	53.8 % (7/13)	46.2 % (6/13)	
Lymph node metastasis				*p* > 0.05
N0	94	38.3 % (36/94)	61.7 (58/94)	
N1	64	37.5 % (24/64)	62.5 % (40/64)	
N2	39	41 % (16/39)	59 % (23/39)	
N3	29	41.4 % (12/29)	58.6 % (17/29)	
Nuclear grade				*p* < 0.05
1	26	61.5 % (16/26)	38.5 % (10/26)	
2	86	43 % (37/86)	57 % (49/86)	
3	114	30.7 % (35/114)	69.3 % (79/114)	
H3K27me3				*p* > 0.5
Positive	220	39 % (86/220)	61 % (134/220)	
Negative	6	33.3 % (2/6)	66.7 % (4/6)	
Total	226	38.9 % (88/226)	61.1 % (138/226)	

### The association between EZH2 expression and clinicopathologic characteristics of breast carcinoma

**Fig. 1:**
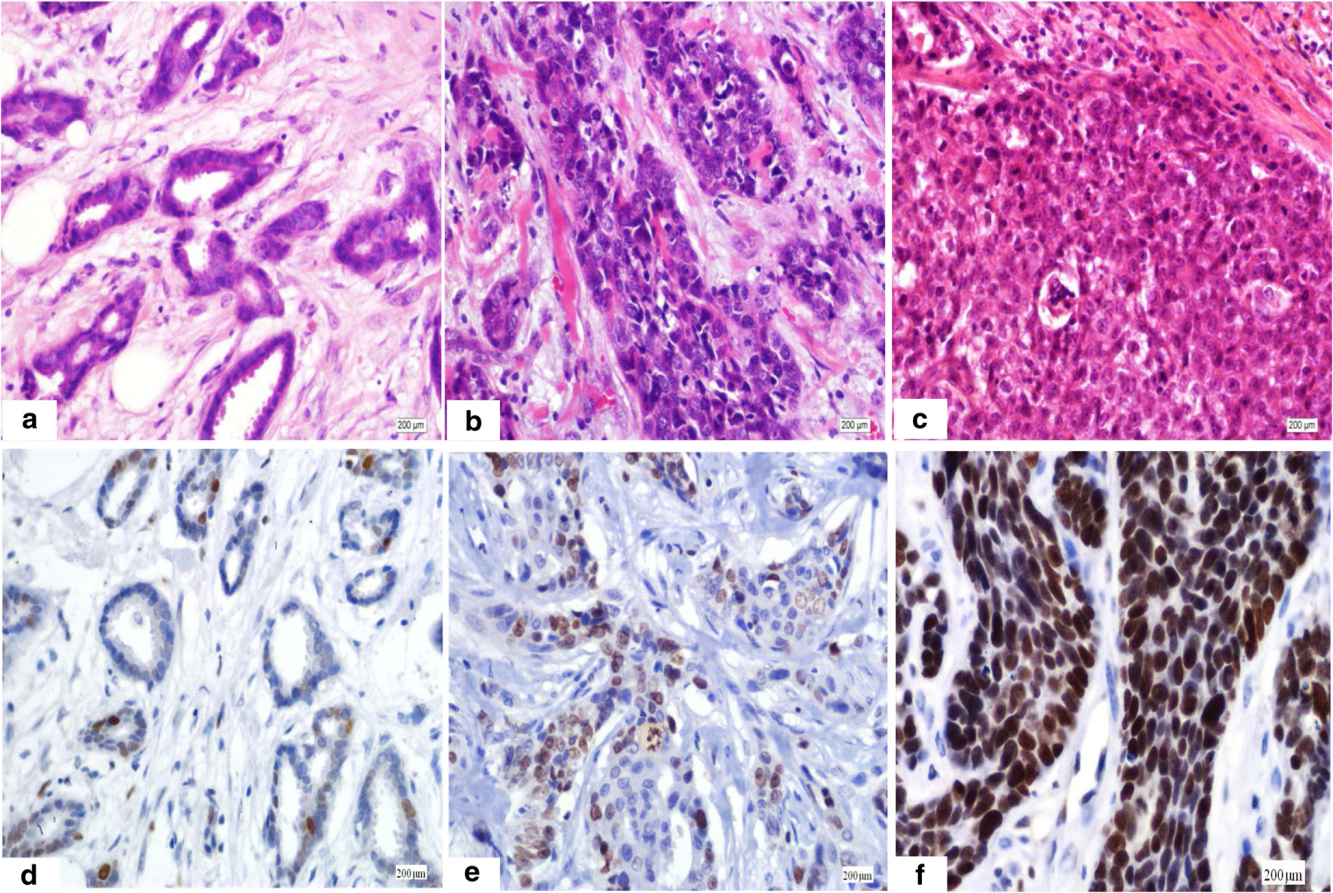
Immunostaining of EZH2 in invasive breast carcinoma of different nuclear grade with variable intensity and quantity were shown. Low grade tubular carcinoma showed bland morphology with well formed tubules and mild atypia (**a**). A low level of EZH2 expression with only scattered positive cells was observed in tubular carcinoma (**d**). Invasive carcinoma, not otherwise specific type of nuclear grade 2 (**b**) with moderate atypia showed moderate expression of EZH2 (**e**). Poorly differentiated carcinoma of nuclear grade 3 was characterized with obvious atypia and solid growth pattern (**c**). Homogeneously strong expression of EZH2 was detected in poorly differentiated carcinoma (**f**). (Magnification × 100)

Of 226 cases of breast carcinoma, 209 cases of breast carcinoma showed nuclear immunostaining of EZH2 with variable intensity and quantity. Low grade tubular carcinoma with well formed tubules and mild nuclear atypia (Fig. 1a) showed a low level of EZH2 expression with only scattered positive cells (Fig. 1d). Invasive carcinoma, not otherwise specific type of nuclear grade 2 (Fig. 1b) showed moderate expression (Fig. 1e). Poorly differentiated carcinoma of nuclear grade 3 with obvious atypia and solid growth pattern (Fig. 1c) showed homogeneously strong expression of EZH2 (Fig. 1f), while seventeen low grade cases did not show any expression. Of these cases, 61.1 % (138/226) were defined as EZH2-overexpressing with a multiplicative score of intensity and quantity > 3. Expression of EZH2 was inversely related to the statuses of ER and PR and significantly higher (Chi-square, *p* < 0.001) in ER- and PR-negative cases; the frequency of EZH2 overexpression in ER- and PR-positive cases was 42.1 % (51/121) and 43.6 % (48/110), versus 82.9 % (87/105) and 77.6 % (90/116) in ER- and PR-negative cases respectively. Overexpression of EZH2 was significantly higher in HER2-positive cases than HER2-negative, 76 % (38/50) versus 56.8 % (100/176) (Chi-square, *p* < 0.05). EZH2 overexpression was significantly associated with the proliferative index, with 25.7 % (19/74), 68.5 % (50/73), and 87.3 % (69/79) of breast carcinomas having overexpression with a low (Ki67 < 14 %), moderate (Ki67 14 ~ 30 %) or high (Ki67 ≥ 30 %) proliferative index, respectively (Chi-square, *p* < 0.001). The correlation between EZH2 overexpression and nuclear grade was statistically significant, with 38.5 % (10/26), 57 % (49/86), and 69.3 % (79/114) of grade 1, 2, and 3 breast carcinomas having overexpression of EZH2, respectively (Chi-square, *p* < 0.05). Intriguingly, none (0/15) of the specific-type low-grade carcinomas (10 tubular carcinomas, five mucinous carcinomas) had EZH2 overexpression. No statistically significant difference was observed for other clinicopathologic features including age, menstrual status, tumor stage (pT), lymph node stage (pN) and expression of H3K27me3. The association of EZH2 expression and clinicopathologic characteristics of breast carcinoma is shown in Table 2. Single, small clusters of carcinoma cells were obscured in the stroma, and lobular cancerization was strongly evidenced by nuclear expression of EZH2 in some cases.

### EZH2 expression in different immunophenotype of breast carcinoma

**Fig. 2:**
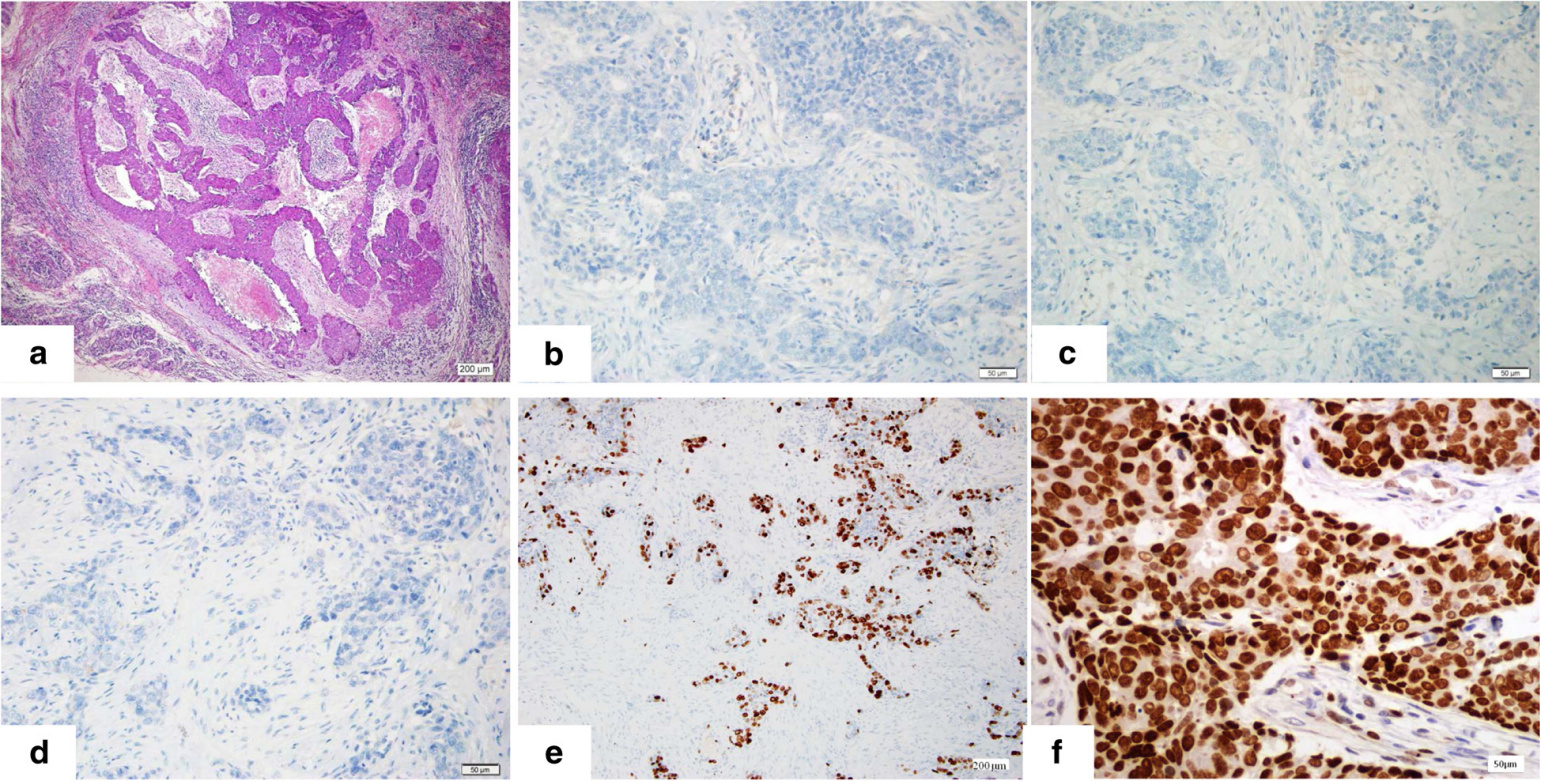
Triple-negative breast carcinoma showed diffusely strong positivity for EZH2. Typical histologic features such as high grade tumor cells arranged in cords and nest with prominent necrosis and pushing border were shown in HE stained section (**a**, Magnification × 100). The tumor cells were negative for HER2, ER, and PR (**b**, **c**, **d**, Magnification × 400), and showed high tumor proliferative index Ki67 (**e**, Magnification × 100). Strong overexpression of EZH2 was observed in high grade triple-negative breast carcinoma (**f**, Magnification × 400)

According to the statuses of ER, PR, HER2 and Ki67, all breast carcinomas were classified into four subtypes: ER-positive with a low proliferative index (Ki67 < 14 %), ER-positive with a moderate to high proliferative index (Ki67 ≥ 14 %), HER2 positive, and triple-negative. Of the 226 cases, 121 were classified as ER positive with at least 1 % positive tumor nuclei in the section. Of these, 54 cases were ER-positive with a low Ki67 index and 67 cases were ER-positive with a moderate to high Ki67 index. Fifty cases were regarded as HER2-positive either by demonstrating overexpression of the HER2 protein using IHC or *HER2* gene amplification by FISH. The remaining fifty-five cases did not express either ER or PR nor showed overexpression of the HER2 protein or *HER2* gene amplification; these were classified as triple-negative. ER-positive breast carcinoma with a low proliferative index showed the lowest percentage of EZH2 overexpression, whereas triple-negative breast carcinoma showed the highest percentage of EZH2 overexpression, 18.5 % (10/54) versus 90.9 % (50/55), (Chi-square, *p* < 0.001). The percentage of EZH2 overexpression in ER-positive breast carcinomas with a moderate to high proliferative index and HER2 positive groups were 61.2 % (41/67) and 74 % (37/50) respectively. Intriguingly, EZH2 was uniformly strongly overexpressed in 88 % (44/50) of triple-negative breast carcinomas with the maximum multiplicative score of 9. Representative case of triple-negative breast carcinoma showed typical histologic features such as high grade tumor cells arranged in cords and nests with prominent necrosis and pushing border was shown in HE stained section (Fig. 2a). The tumor cells were negative for HER2, ER, and PR (Fig. 2b, c, d), and triple negative breast carcinoma usually showed high tumor proliferative index Ki67 (Fig. 2e). Strong overexpression of EZH2 with a score of 9 was observed in high grade triple-negative breast carcinoma (Fig. 2f).

**Fig. 3:**
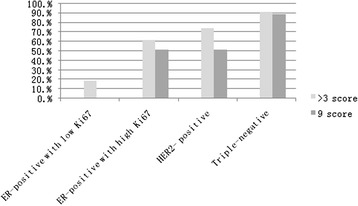
EZH2 overexpressed in breast carcinoma and association with immunophenotype. Triple- negative breast carcinoma showed the highest percentage of EZH2 overexpression: 90.9 % (50/55) were defined as EZH2 overexpression with a multiplicative score of intensity and percentage > 3, and 88 % (44/50) among them showed strong overexpression of EZH2 with a maximum multiplicative score of 9. Low-grade ER-positive cases with low Ki67 index (<14 %) showed the lowest percentage of EZH2 overexpression, with only 18.5 % (10/54) having a multiplicative score >3, and no case garnered a maximum multiplicative score

The sensitivity and specificity of EZH2 overexpression for detecting triple-negative was 51 and 86 % respectively. EZH2 expression in ER-positive cases with moderate to high proliferative index or HER2 positive breast carcinoma was variable in both intensity and quantity, and 51.2 % (21/41) and 51.4 % (19/37) showed strong EZH2 overexpression with a maximum score of 9 in these two cohorts respectively. No case of ER-positive breast carcinoma with a low proliferative index showed strong overexpression of EZH2 with a score of 9 (Table 3 and Fig. 3).

**Table 3:** Expression of EZH2 in subtype of breast carcinoma

Clinicopathologic features		EZH2 score		
	Total N	>3 score, positive	9 score, positive	*P* value
ER positive with low Ki67	54	18.5 % (10/54)	0 % (0/10)	*p* < 0.001
ER positive with high Ki67	67	61.2 % (41/67)	51.2 % (21/41)	
Her2 positive	50	74 % (37/50)	51.4 % (19/37)	
Triple-negative	55	90.9 % (50/55)	88 % (44/50)	
Total	226	61.1 % (138/226)	62.3 % (86/138)

### EZH2 overexpression in high-grade DCIS adjacent to triple-negative breast carcinoma

**Fig. 4:**
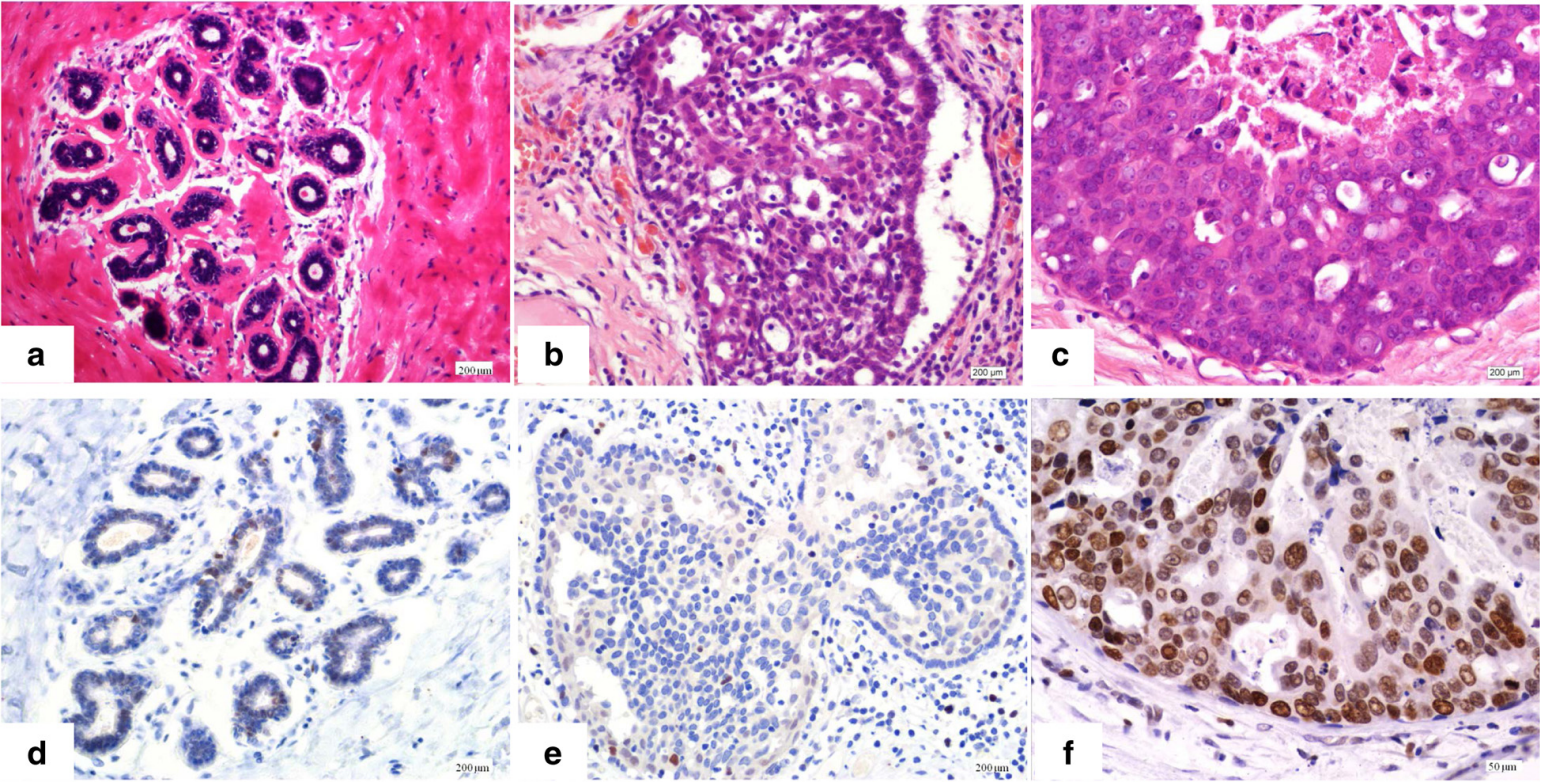
Immunostaining of EZH2 in benign lesions and high grade DCIS associated with triple-negative invasive breast carcinoma. Breast lobules, and UDH which showed benign proliferation of epithelial and myoepithelial cells without any atypia were shown (**a** and **b**). Breast lobules, and UDH expressed EZH2 in a low level, and only occasional EZH2 positive cells were observed (**d** and **e**). Representative high-grade DCIS associated with invasive triple-negative breast carcinoma showed solid growth pattern and central necrosis (**c**) (Magnification × 100). EZH2 was strongly overexpressed in high grade DCIS (**f**) (Magnification × 400)

EZH2 was mainly observed in the nuclei of epithelial cells with variable intensity and quantity in breast tissue. There was weak immunostaining of EZH2 in stromal fibroblasts, whereas the infiltrative germinal center B cells of lymphoid follicles showed homogeneously strong positivity. The expression of EZH2 in 20 benign breast lesions comprising of adenosis and usual ductal hyperplasia (UDH) was also assessed. Compared with normal or benign hyperplastic lobules and ducts, EZH2 expression was elevated in high-grade DCIS adjacent to triple-negative breast carcinoma (0 versus 90 %, *p* < 0.001) (Table 4). Breast lobules and UDH (Fig. 4a, b) only expressed EZH2 in a low level (Fig. 4d, e), whereas EZH2 was strongly overexpressed in high-grade DCIS (Fig. 4c, f), associated with invasive triple-negative breast carcinoma, and homogeneously strong positivity in 90 ~ 100 % cells was observed; however, expression of EZH2 in low-grade DCIS was variable, with 55 % cases showing overexpression. The intensity and quantity of EZH2 expression in DCIS was identical to those of corresponding invasive components of the same case on the same biopsy.

**Table 4:** Expression of EZH2 in benign lesions and associated with invasive carcinoma

Pathologic feature		EZH2 score		
	Total N	≤3	>3	*p* value
Adenosis	10	100 % (10/10)	0	*p* < 0.001
UDH	10	100 % (10/10)	0	
DCIS associated with high grade triple-negative carcinoma	20	10 % (2/20)	90 % (18/20)	
DCIS associated with low to moderate grade non-triple-negative carcinoma	20	45 % (9/20)	55 % (11/20)

## Discussion

Breast carcinomas are extraordinarily heterogeneous with different clinicopathologic features and outcomes. The statuses of ER, PR and HER2 are important for treatment and prognosis. ER positive and HER2 positive breast carcinoma can benefit from anti-endocrine and anti-HER2 therapy, respectively. However, triple-negative carcinoma shows aggressive behavior but do not benefit from current available anti-endocrine or anti-HER2 targeted molecular therapy [3–5]. Triple-negative breast carcinoma is defined by immunophenotypic negativity for ER, PR and HER2 [3–5]. Without a targeted molecular therapy, triple-negative breast cancer will continue to be threatening, thus identifying potential targets will inform efforts to combat this aggressive disease.

In the present study, we found that EZH2 was significantly overexpressed in triple- negative breast carcinoma and was associated with high tumor cell proliferation, whereas low grade ER-positive breast carcinoma such as tubular carcinoma and mucinous carcinoma showed low EZH2 expression levels. A significant association between EZH2 overexpression with features of aggressive breast carcinoma including high nuclear grade and HER2 positivity was also found.

EZH2 expression was previously evaluated in breast carcinomas and was reported to be significantly associated with increased tumor cell proliferation and as a marker of aggressive behavior [21, 22]. The prognosis of EZH2-positive breast carcinoma is worse than that of EZH2-negative cases, as the 5-year survival rate was 81 and 91 % in patients with EZH2-positive or -negative breast carcinoma, respectively [21]. As overexpression of EZH2 in immortalized human mammary epithelial cell lines promotes anchorage-independent growth and cell invasion, it is possible that EZH2 is involved in tumorigenesis of breast carcinoma [22]. We found that EZH2 is significantly overexpressed in high-grade triple-negative breast carcinoma: 90.9 % (50/55) of triple-negative breast carcinoma showed overexpression of EZH2 with a multiplicative score of intensity and percentage >3, and 88 % (44/50) among them showed homogeneously strong overexpression in 90 ~ 100 % tumor cells with a multiplicative score of 9 (Table 2). Conversely, ER-positive low grade breast carcinoma with low proliferation (Ki67 < 14 %) showed the lowest level expression of EZH2, with only 18.5 % (10/54) overexpressing EZH2; no case in this group showed diffuse strong overexpression (Table 3).

We found that EZH2 expression in breast carcinoma was significantly associated with tumor cell proliferation: 87.3 % (69/79) of breast carcinoma with high Ki67 index (>30 %) showed high EZH2 levels, whereas only 25.7 % (19/74) invasive breast carcinoma with a low Ki67 index (<14 %) showed EZH2 overexpression. As EZH2 also regulates cell cycle in addition to its histone H3 methyltransferase function [23], and EZH2 overexpression contributes to uncontrolled mammary cell proliferation [24], the significant association between EZH2 overexpression and Ki67 proliferative index further provides evidence of EZH2 in the regulation of breast tumor cell proliferation. The association between expression of EZH2 and HER2 is controversial in literature [9, 22]. We also found that EZH2 overexpression was significantly high in HER2 positive breast carcinoma by IHC or FISH comparing with negative cases, 76 % (38/50) versus 56.8 % (100/176), *p* < 0.05. The significant association between EZH2 overexpression and HER2 also supports a role of EZH2 in regulation of tumor cell proliferation.

Recently, it was reported that *BCL11A* is a novel triple-negative breast carcinoma oncogene, which was expressed in 66.7 % (16/24) of triple-negative breast carcinomas [25]. We did not evaluate BCL11A expression in our 226-case panel. Still, a direct comparison between BCL11A and EZH2 in a large cohort is necessary to decide if one better prognosticates. Further, we show that EZH2 overexpression tracks with independently verified immunohistologic identification in our 226-case study on breast carcinoma, particularly identifying essentially all triple-negative cases and a large proportion of high-grade HER2-positive cases. Consistent with previous studies [26, 27], we observed that EZH2 expression in normal epithelial cells of the terminal ductal lobular unit of the breast and cells of benign breast lesions is low with a multiplicative score <3. This suggests that low levels of EZH2 are necessary to regulate normal mammary epithelial cell proliferation; however, EZH2 overexpression in ER-negative MDA-MB-231 cells results in decreased BRCA1 and uncontrolled proliferation which contributes to tumorigenesis of breast carcinoma [24]. Intriguingly, we observed that EZH2 was strongly overexpressed in high-grade DCIS adjacent to triple-negative breast carcinoma, whereas UDH in benign lesions did not overexpress EZH2. This suggests that aberrant expression of EZH2 may occur in the early stage of carcinogenesis. Previous studies revealed that EZH2 was up-regulated in DCIS and even morphologically normal breast epithelial cells from patients with increased risk of breast carcinoma [26, 27], and EZH2 was reported as a molecular marker for identifying a precancerous state in morphologically normal breast, but these studies did not reveal the relationship of EZH2 and triple-negative breast carcinoma and its *in situ* components [26, 27]. Our finding of high overexpression of EZH2 in triple-negative DCIS as well as invasive breast carcinoma supports an important role of EZH2 in carcinogenesis of triple-negative breast carcinoma. Furthermore, the highest level of EZH2 expression was observed in high nuclear grade triple-negative DCIS, which is consistent with the clinical evolution of DCIS, as it is widely accepted that high nuclear grade DCIS is associated with higher rate of recurrence than low and intermediate nuclear grade DCIS. The finding suggests that the high expression of EZH2 protein is associated with breast carcinoma progresses. As we found that EZH2 expression is unrelated to the status of H3K27me3 in the present study (Table 2), we argue it is likely that EZH2 works in a histone methyltransferase-independent manner in breast carcinoma pathogenesis. We think EZH2 may further prove to be a useful marker for high-grade triple-negative DCIS. Identification of high-grade DCIS could be clinically meaningful and likely necessitates close follow-up of these patients. Furthermore, as nuclear staining of EZH2 can display single or small cluster of tumor cells embedded in stroma, it is useful in detecting micro-invasion of breast carcinoma in some cases.

The reason that epithelial cells lose ER and PR without HER2 amplification during the process of malignant transformation in triple-negative breast carcinoma, however, remains unclear. It is reported that EZH2 forms a complex with NF-κB and activates transcription activity of NF-κB in ER-negative MDA-MB-231 cells [24, 28]. As this study demonstrates that EZH2 is consistently overexpressed in high grade triple-negative breast carcinoma, we hypothesize that EZH2 plays an important role in tumorigenesis for this unique breast carcinoma.

## Conclusion

In summary, our study demonstrates that EZH2 overexpression is significantly associated with high grade triple-negative breast carcinoma as well as corresponding DCIS, plus many high-grade HER2-positive breast carcinomas. Because there was a strongly high association between the Ki67 proliferation index and EZH2 expression levels, we argue that EZH2 overexpression likely confers a proliferative advantage to EZH2-high cells which allows these tumor cells to escape cell cycle regulation. Thus, inhibiting EZH2 function may be relevant for treatment of triple-negative breast carcinoma, which currently has no targeted, effective therapy. Perhaps the next step is to assess the utility of EZH2 inhibitors in EZH2-overexpressing triple-negative breast carcinomas. EZH2 is a promising target for prognostication and treatment in the aggressive and otherwise unresponsive triple-negative breast carcinomas.
